# Accuracy of the One-Stage and Two-Stage Impression Techniques: A Comparative Analysis

**DOI:** 10.1155/2016/7256496

**Published:** 2016-11-24

**Authors:** Ladan Jamshidy, Hamid Reza Mozaffari, Payam Faraji, Roohollah Sharifi

**Affiliations:** ^1^Department of Prosthodontics, School of Dentistry, Kermanshah University of Medical Sciences, Kermanshah, Iran; ^2^Department of Oral Medicine, School of Dentistry, Kermanshah University of Medical Sciences, Kermanshah, Iran; ^3^Student Research Committee, School of Dentistry, Kermanshah University of Medical Sciences, Kermanshah, Iran; ^4^Department of Endodontics, School of Dentistry, Kermanshah University of Medical Sciences, Kermanshah, Iran

## Abstract

*Introduction*. One of the main steps of impression is the selection and preparation of an appropriate tray. Hence, the present study aimed to analyze and compare the accuracy of one- and two-stage impression techniques.* Materials and Methods*. A resin laboratory-made model, as the first molar, was prepared by standard method for full crowns with processed preparation finish line of 1 mm depth and convergence angle of 3-4°. Impression was made 20 times with one-stage technique and 20 times with two-stage technique using an appropriate tray. To measure the marginal gap, the distance between the restoration margin and preparation finish line of plaster dies was vertically determined in mid mesial, distal, buccal, and lingual (MDBL) regions by a stereomicroscope using a standard method.* Results*. The results of independent test showed that the mean value of the marginal gap obtained by one-stage impression technique was higher than that of two-stage impression technique. Further, there was no significant difference between one- and two-stage impression techniques in mid buccal region, but a significant difference was reported between the two impression techniques in MDL regions and in general.* Conclusion*. The findings of the present study indicated higher accuracy for two-stage impression technique than for the one-stage impression technique.

## 1. Introduction

The impression process includes careful transfer of the patient's soft and hard tissues to laboratory and is a major part of fixed prosthetic treatments. Since the patient's soft and hard tissues are transferred, having anatomic knowledge about periodontal tissues, making an accurate impression especially in the finish line, and using proper impression materials and an appropriate impression technique are important in making a suitable and accurate impression [1]. The impression technique determines the restoration of finish line. Moreover, the significance of margin in the longevity of restoration and the effect of impression technique on marginal adaptation of restoration indicate the necessity of applying an accurate impression technique. The accuracy of impression techniques is revealed when restoration with suitable marginal adaptation and minimum gap is obtained [2]. The mechanical and bonding characteristics [3] are also significantly influenced by the marginal fit [4–12]. Inaccurate marginal fit causes plaque accumulation, microleakage, and cement breakdown. Subsequently, the risk of carious lesions, periodontal disease, and endodontic inflammation is increased, and adverse consequences that affect the underlying health of abutments may occur [4–10].

Several studies have evaluated the maximal marginal gap values [13–20]. McLean evaluated more than 1000 crowns in 5 years and concluded that a marginal gap of less than 120 *µ*m is clinically acceptable [13]; however, in vitro studies have reported values of 100 *µ*m [15–20]. In addition to impression techniques, various factors, including preparation finish line, preparation angle, type of die, type of sprue, dye spacer [2], type of alloy and casting method, and type of impression material, influence the marginal adaptation [21]. Nowadays, there are various impression materials for casting restorations that are accurate enough. In general, the common impression materials include hydrocolloid and elastomeric impression materials. They have special properties of their own and their selection depends on the existing factors and conditions [22]. In cases where there is not enough time for pouring the impression, polyether and additional silicone are used because these materials have high dimensional stability and their impression can be kept for a long time [23]. Additional-polymerization silicone, also known as polyvinyl siloxane, was first introduced as an impression material in the 1970s. Additional silicones are very much similar to condensation silicone, except that additional silicone has higher dimensional stability (its dimensional stability is the same as polyether). The working time of additional silicone is greatly influenced by the ambient temperature and the hardened material has lower rigidity than polyether. Additional silicone is one of the most accurate and stable impression materials, which is used as single-paste, double-paste, and putty-wash systems [23, 24]. Currently, the putty-wash type is widely used in fixed prosthodontics and removable overdentures. This technique does not require a special tray and the putty-wash impression plays the role of special tray and saves time [25]. Moreover, there are two techniques for double-stage impression, impression with spacer and impression without spacer, and the latter was used in the present study. Based on the abovementioned discussion and diverse ideas about impression techniques, the current research was conducted to evaluate and compare the accuracies of one- and two-stage impression techniques.

## 2. Materials and Methods

A standard preparation was performed on the mandibular molar model with the following characteristics (W&H turbine, Allegra): 1.5 mm occlusal reduction and 1 mm axial reduction with 6–8° convergence (3-4° for each side) [7]. Round shoulder finishing line with at least 1 mm width was prepared on the lingual and facial surfaces, which were reduced in two planes [5, 20], and all of the line angles were rounded to reduce stress concentrations. Then, impression making was performed 20 times with one-stage impression technique and 20 times with two-stage impression with a proper tray using panacil additional silicone (Ketten Bach, Germany). Using adhesive plastic strips, stops were created in the tray to prevent sitting tray on the teeth and to provide sufficient space for impression making. The stops were placed at the posterior and anterior parts of the tray [24, 26].

In one-stage impression technique, putty and wash were mixed simultaneously, putty was placed in the tray, wash was put on the tooth surface, and impression was made with the same applied pressure on the tray in the mouth during impression making [24]. In two-stage impression technique, however, an impression was made with putty from the prepared tooth and interdental papillary regions were removed, afterwards. Then, several vents were created within the impression material for escape. Uniform thickness of material (putty or wash) must be used by applying uniform pressure for all the impression making. If the impression material does not escape, it exerts pressure on the putty, and, after taking the impression out of the mouth, the putty returns to its original place and the impression becomes smaller consequently. Next, the impression was relined again with a layer of wash (3-4 mm) [26] by using uniform thickness of material (putty or wash) by applying uniform pressure for all impressions. The casts of the intended impression were poured with plaster type IV over 30 minutes according to the manufacturer's instructions (first plaster). Then, the second part of plaster was added half an hour later using stone type III. To prepare the plaster, the plaster powder was slowly added to the water container during a period of 10 seconds and was mixed by vacuum mixer for 30 seconds [2] to minimize the bubbles. The prepared gypsum was poured in the impression on the vibrator over 3 minutes, and, one hour later, the gypsum model was extracted from the cast and randomly coded by another person [21].

The plaster dies with bubble at the die site and preparation finish line were excluded from the study, so a total of 40 plaster models were obtained. All of the dies were covered with two layers of spacer with resultant thickness of 30 *µ*m. Care must be taken that the varnish is 0.5 mm short of finish line [11]. To make the metal copings, the construction procedures were performed on the dies. Investing and casting were done for all samples in similar laboratory conditions. After casting the metal coping, the sprue was cut and coping was seated on the dies by using fit checker and eliminating the premature points on the intaglio surface of coping. To measure the marginal gap, the distance between the copings margin and preparation finish line of plaster dies (master cast) was determined vertically in mid mesial, distal, buccal, and lingual regions by a stereomicroscope.

## 3. Results 

**Figure 1:**
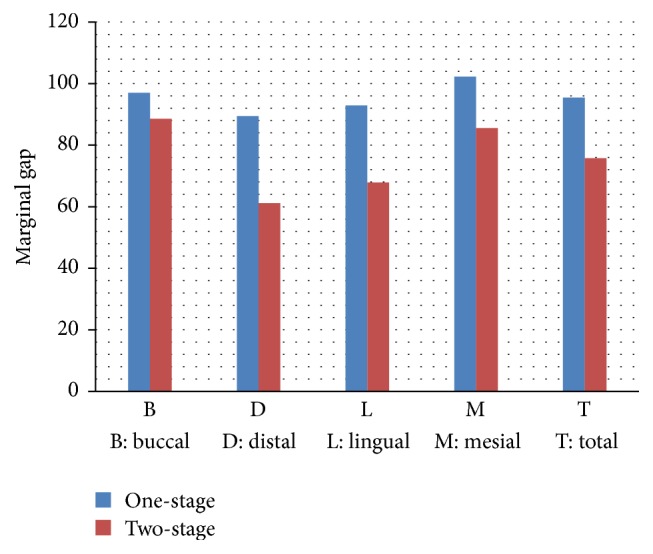
Comparison of the means of marginal gaps for one- and two-stage impression techniques.

Before running *t*-test with two independent variables, Kolmogorov-Smirnov test was used to analyze the normality of variables and the results confirmed that the data were normally distributed (*p* > 0.05). Having ensured the normality of variables, independent *t*-test was applied and the findings showed no significant difference in marginal gap between one- and two-stage impression techniques in mid buccal region (*p* > 0.05). However, a significant difference was reported between the two impression techniques in mid mesial, distal, and lingual regions and average of all surfaces (*p* < 0.05). The results of independent *t*-test are shown in Table 1. Detailed diagram of the marginal gap in mesial, lingual, distal, and buccal side in the one- and two-stage impression techniques is demonstrated in Figure 1.

## 4. Discussion

As mentioned, making impression for oral restoration and dental morphology is an integral part of prosthodontics, and accurate impression is undoubtedly one of the most important stages of fixed prosthetic treatments. Ignoring this treatment stage will result in inaccurate impression and consequently a prosthesis with improper adaptation. Lack of accuracy in impression making leads to repeated impression making, which is costly and time-consuming for the patient. Thus, selecting the best and most accurate impression technique seems to be necessary for a successful treatment [1, 2].

One- and two-stage impression techniques are acceptable for many clinicians, and no significant difference has been reported in most of the studies [2]. Therefore, given the diversity of ideas about the impression technique, the effect of various factors on the treatment outcome, and the notion that application of the most accurate impression technique requires repeated impression in special cases due to inaccurate preparation finish line, the current research was conducted to evaluate and compare the accuracies of one- and two-stage impression techniques. In most in vitro studies similar to the present study, the prepared and standardized dies have been desirably used to evaluate the accuracy of copings [27], since 40 gypsum dies were made from one dental model, and precise control was exerted on preparation parameters like convergence rate of axial walls and preparation finish line. Hence, the impression technique variable was evaluated and compared and the rest of the variables were kept fixed and under control. Also, sectioning technique was not used in the current study, which might have caused marginal distortion and reduced accuracy of measurement due to heat production [27].

The results of this study revealed that the marginal gap in the fabricated copings by one- and two-stage impression techniques was less than 120 *µ*m, which is clinically acceptable according to some studies [14–20]. Better marginal adaptation at the margins can decrease the rate of fracture by increasing the consistency and can decrease microbial plaque and periodontal disease and complications consequently. Therefore, the copings made by one- and two-stage impression techniques are recommended to be utilized.  Moreover the mean marginal gap is lower in the copings of two-stage impression. Therefore, this technique is preferred to one-stage impression technique, which is in line with the results of the studies conducted by Mahshid et al. [28], Caputi and Varvara [29], Nissan et al. [30], and Dugal et al. [31].

In their studies, Franco et al. reported no significant difference between one-stage impression technique and impression technique with a spacer [32]. The result of this study was similar to the result of Franco's study [32], but in this study we did not use a spacer. This subject probably was due to impression making with the spacer which is induced impression material remaining in the tray, with more accurate impression making than without a spacer.

Vitti et al. evaluated the dimensional accuracy of stone casts based on the impression material and three impression techniques. They found that stone casts had high dimensional accuracy, and one-stage and two-stage putty-wash impression techniques and monophase light-body impression technique were not significantly different for marginal gap [33]. The difference between the findings of Vitti et al.'s study and those of the current study can be due to the type of impression material. Vitti et al. used two types of materials: silicone and gypsum; furthermore, we use only gypsum material and this may make difference in results.

However, better marginal adaptation may be associated with proper thickness of wash in this study with proper pressure by applying 10 newtons during impression making, which may have made up for the putty condensation and reduced the pressure on putty impression in the second stage, thereby minimizing the intensity of condensation resulting from the return to the initial state. No significant difference between one- and two-stage impression techniques in mid buccal region may be associated with buccal direction of thumb finger pressure above tray during impression making, which may dislodge impression tray from other sites (mesial, distal, and lingual), which is compensative in two-stage technique with wash, but there is no fortunate compensation in the one-stage technique. Buccal direction during impression making induces tray dislodgement in other surfaces such as mesial, distal, and lingual regions, which forms a greater marginal gap. In two-stage impression making, the marginal gap is compensated by wash material in the second stage, whereas, in one-stage, we do not have the second stage for compensating marginal gap in such surfaces.

## 5. Conclusion

The findings of this study indicated no significant difference between one- and two-stage impression techniques in mid buccal region (*p* > 0.05), but a significant difference was observed between the two impression techniques in mid mesial, distal, and lingual regions and in average four surfaces (*p* < 0.05). Therefore, it can be argued that the accuracy of two-stage impression technique was higher than that of one-stage impression technique. However, marginal gaps in both techniques were clinically acceptable (marginal gap is less than 120 *µ*m) and suggested.

## Limitations and Suggestions

Since the present study was performed in a laboratory, it was not possible to analyze the effect of such factors as blood, saliva, oral temperature, and special clinical conditions on the accuracy of impression techniques, which might have significant impacts on the obtained results; for example, high temperature in the oral cavity which is due to some hot foods may produce dimensional changes in coping special margins, because coping in this region is so thin, and this change may influence vertical gap. Hence, future studies are recommended to examine this issue in clinical conditions and with different impression materials.

## Figures and Tables

**Table 1:** Descriptive statistics and significance level of independent *t*-test.

Marginal gap	Mesial	Lingual	Distal	Buccal	Total average
One-stage					
Mean ± SD	102.22 ± 66.66	92.95 ± 20.64	89.5 ± 34.10	97.02 ± 33.80	95.42 ± 16.09
Two-stage					
Mean ± SD	85.52 ± 25.27	67.82 ± 31	61.15 ± 26.33	88.55 ± 23.47	75.76 ± 23.47
*p* value	0.028	0.005	0.006	0.360	0.001

^**∗**^SD: standard deviation.
